# General Anesthesia by Hypodermic Method

**Published:** 1907-09

**Authors:** 


### GENERAL ANESTHESIA BY HYPODERMIC METHOD.

The rapidity with which the Abbott-Lanphear method of anesthesia has advanced in the confidence of the profession is unparalleled. The method is simple, easily used, requires less assistants in surgical operations, is acceptable to the patient, is remarkably free from danger; is devoid of after effects to a greater extent,than inhalation anesthesia, is recovered from promptly and can be adjusted to nearly all patients. Its most marked influence is in the slowing of the respiration, which is apt to alarm those who have not proven that no harm results, the respitation being that of deep sleep. The use of hyoscine instead of scopolamine is a great improvement on the original method, and the introduction of this substance, by Dr. Abbott, and of cactus in the form of cactin in the compound, hyoscine, morphine and cactin comp. (H. M. C. Abbott) has added a safeguard which is invaluable, and which in time will be fully appreciated. Abbott has the confidence of the profession because he makes good. On his presentation it was promptly tried and another success is scored.

